# Effect of Fresh Orange Juice Intake on Physiological Characteristics in Healthy Volunteers

**DOI:** 10.1155/2014/405867

**Published:** 2014-03-04

**Authors:** Sedigheh Asgary, Mahtab Keshvari, Mohammad Reza Afshani, Masoud Amiri, Ismail Laher, Shaghayegh Haghjooy Javanmard

**Affiliations:** ^1^Isfahan Cardiovascular Research Center, Isfahan Cardiovascular Research Institute, Isfahan University of Medical Sciences, Isfahan 8187698191, Iran; ^2^Physiology Research Center, Isfahan University of Medical Sciences, Isfahan 7346181746, Iran; ^3^Department of Cardiology, School of Medicine, Isfahan University of Medical Sciences, Isfahan 7346181746, Iran; ^4^Social Health Determinants Research Center and Department of Epidemiology and Biostatistics, School of Health, Shahrekord University of Medical Sciences, Shahrekord 8815713361, Iran; ^5^Department of Pharmacology & Therapeutics, Faculty of Medicine, University of British Columbia, Vancouver, Canada V6T 1Z3

## Abstract

*Background*. Impaired endothelial function is a predictor of cardiovascular events. Orange juice (OJ) is rich in dietary flavonoids and could inhibit oxidative stress and inflammatory responses. We examined the effects of commercial (COJ) and fresh orange juice (FOJ) on endothelial function and physiological characteristics in healthy humans. *Materials and Methods*. Twenty-two healthy volunteers years were enrolled in a single blind randomized crossover controlled trial. The two groups consumed either COJ for the first 4 weeks and then FOJ (CFOJ, 4 weeks), or FOJ for the first 4 weeks and then COJ (FCOJ, 4 weeks). We assessed endothelial function by measuring flow-mediated dilation, serum concentrations of lipids, apolipoproteins A and B (apo A-1 and apo B), and inflammatory markers such as vascular endothelial adhesion molecule 1 (VCAM-1), E-selectin, high-sensitivity C-reactive protein (hs-CRP), and interleukin-6. *Results*. Consumption of both juices decreased VCAM, hs-CRP, and E-selectin but increased apo A-1. A decline in LDL occurred in the FOJ group. There were no differences between the characteristics of two groups, with the exception of apo A-1 levels that were increased with both forms of OJ. The largest variations occurred with hs-CRP, VCAM in both groups. *Conclusion*. Consumption of COJ and FOJ produced beneficial effects on the physiological characteristics of healthy volunteers. Although these results could encourage the consumption of OJ, intervention studies are needed to determine the long-term effects of these types of OJ on metabolic and cardiovascular endpoints.

## 1. Introduction

Cardiovascular diseases (CVD) represent the leading causes of death [1–3]. Nitric oxide (NO) has important role in maintaining cardiovascular homeostasis, such that reduced NO bioavailability precedes atherosclerosis and CVD [4, 5]. Endothelial function, as measured by NO dependent flow-mediated dilation (FMD), is a predictor of cardiovascular events [6, 7]. Conditions such as insulin resistance, diabetes, and diet affect FMD [8]. Inflammation as well as low NO plays key roles in the activation and progression of atherosclerosis [9, 10].

Epidemiologic studies suggest that the Mediterranean diet, which is rich in antioxidants found in fresh fruit and vegetables, is associated with a reduced risk of chronic diseases such as cancer and coronary artery disease [11–15]. Furthermore, epidemiologic studies consistently report protective effects of polyphenol-rich foods (fruit, tea, wine, cocoa, chocolate, and especially citrus fruit) on some intermediate risk factors for CVD such as LDL cholesterol, blood pressure (BP), and endothelial function [16–18].

The reduction of oxidative and inflammatory stress by orange juice (OJ) is partially attributed to two flavonoids, hesperidin and naringenin, through decreasing atherogenic LDL cholesterol in hypercholesterolemic (HCH) and increasing HDL ability to take up free cholesterol in HCH and normolipidemic (NC) which may be beneficial for cholesterol esterification and reverse cholesterol transport; in addition, caloric intake in the form of OJ or fructose may not induce either oxidative or inflammatory stress, possibly due to its flavonoids content, and might represent a potentially safe energy source [19, 20]. The high global consumption of OJ has resulted in the increased availability of frozen concentrated orange juice to the extent that the market share of this product exceeds that of the fresh fruit. Compared to fresh orange juice, concentrated (commercial) OJ has a greater flavonoid content of polymethoxylated flavones (such as hesperidin) and pectin and essential oils (from the peel) due to the manufacturing process which uses the entire fruit to produce juice [21]. In fact, OJ may decrease diastolic blood pressure (DBP) when regularly consumed and postprandially increases endothelium-dependent microvascular reactivity as well as the possibility that hesperidin could be causally linked to the beneficial effect of OJ [22].

We hypothesized that the intake of commercial (COJ) and fresh (FOJ) orange juice reduces markers of inflammatory stress and so produces short-term favorable effects on endothelial function of healthy volunteers as measured flow-mediated dilation (FMD), serum concentrations of lipids, apolipoproteins A and B (apo A-1 and apo B), and inflammatory markers such as vascular endothelial adhesion molecule 1 (VCAM-1), E-selectin, high-sensitivity C-reactive protein (hs-CRP), and interleukin-6 (IL-6).

## 2. Methods

Twenty-two healthy volunteers (7 men and 15 women) aged 18–59 years, with no evidence of chronic, metabolic, or endocrine diseases, were invited to participate in a single-blind randomized crossover controlled clinical trial at the Isfahan Cardiovascular Research Center in Isfahan, Iran, in 2012. After explaining the study design, 22 volunteers agreed to participate in the trial. Exclusion criteria used were the use of medications, antioxidant or vitamin supplements, intense physical activity (more than 5 hours per week), smoking, and vegetarian or other restrictive dietary requirements. The Ethics Committee of Isfahan University of Medical Sciences approved the study protocol and completed consent letters were obtained from all participants. Commercial orange juice concentrate and freshly squeezed orange juice were used to prepare COJ and FOJ daily. The participants consumed orange juice in the presence of study investigators, even on holidays and weekends. Participants were required to submit a food diary for the study period.

Participants were randomly assigned into two equal groups each consisting of 11 volunteers. The volunteers in the first group consumed COJ for the first 4 weeks followed by a 2-week washout period; they then consumed FOJ (CFOJ group). The second group consumed FOJ for the first 4 weeks followed by a 2-week washout period; they consumed COJ (FCOJ group). Throughout the two 4-week periods of FOJ or COJ consumption, subjects consumed 500 mL, twice a day with breakfast and dinner [23, 24]. We purchased COJ (brand name: Takdaneh Company) and ensured that all COJ was of similar composition. Fresh oranges (*Citrus sinensis* Swing brand) were used to extract juice.

**Figure 1:**
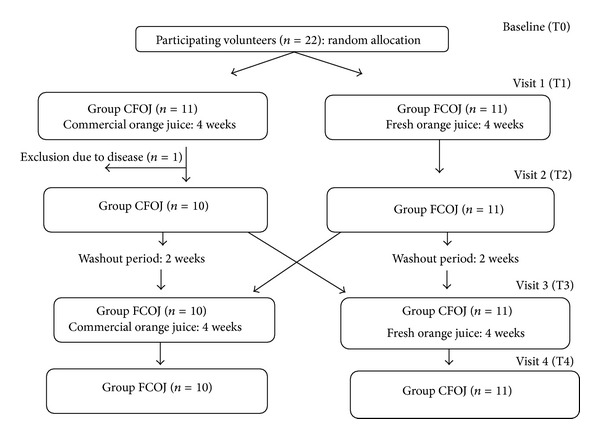
Diagram of randomized crossover clinical trial.

One of the participants in CFOJ group was excluded from the study due to the diagnosis of an underlying disease and this subject was omitted from all data analysis (Figure 1).

There were four visits for each subject: the day before each experiment (visits 1 and 3) and the day after each experiment (visits 2 and 4) where body mass index (BMI) and FMD measurements were made. Body height was measured to the nearest 0.1 cm with the participants barefooted. Body weight was measured to the nearest 0.05 kg with calibrated digital scales (AMZ 14; Mercury, Tokyo, Japan) with the participants wearing light clothing and no footwear. The BMI index was calculated by dividing the weight (kg) by height (m) squared [25].

Measurements of FMD were made in the fasting state and before withdrawal of blood samples. Twelve-hour fasted blood samples (5 mL) were collected from the left antecubital vein between 8:00 and 9:30 a.m. Blood was collected in vacutainer tubes without anticoagulant, under quality control and safety procedures. Serum was separated from blood 2-3 hrs after sampling by centrifugation at 3500–4000 rpm for 10 min. Collected sera were stored at −80°C until analysis. Serum lipid profile (comprising total cholesterol, triglycerides, and low- (LDL-C) and high-density lipoprotein cholesterol (HDL-C)) along with high-sensitivity C-reactive protein (hs-CRP) and fasting blood sugar (FBS) was determined using automated enzymatic assays (Pars Azmoon, Tehran, Iran) on a Hitachi 902 autoanalyzer. Serum concentrations of adhesion molecules including vascular endothelial adhesion molecule 1 (VCAM-1) and E-selectin together with interleukin-6 (IL-6) were measured by enzyme-linked immunosorbent assay (ELISA) kits (Boster Biological Technology, Wuhun, China). Apo B and Apo A-1 concentrations were quantified using a modified commercially available immunoturbidimetric assay according to the kit instructions (Pars Azmoon, Tehran, Iran). Endothelial function as measured by flow-mediated dilation (FMD) in the right brachial artery was determined before and after each ultrasonography and was calculated as the percentage change from baseline diameter [26].

## 3. Statistical Analysis

Statistical analysis was conducted using Proc Mixed from SAS statistical software (version 9.2; SAS Institute Inc., Carry, NC). Measurements at the end of each treatment were considered as response variables in a linear mixed model where baseline measurements were covariates. Using this model, we considered the treatment period and their interactions as fixed effects while subjects assigned as random effects. A significant main effect of treatment was indicated when the *P* value of the *F* test for the main effect was lower than 0.05.

Carryover effects was considered within mixed models and Proc Mixed as well for measurements with missing cases. Carryover effects occurred only for FBS and Apo A-1 and treatment effects were estimated by adjusting for the carryover effect.

We compared the clinical characteristics of the two treatment groups (COJ first and FOJ first) using a paired *t*-test for continuous variables. Each treatment effect (COJ effects on base measurement and FOJ effect on baseline measurements) was tested using a paired *t*-test for continuous variables (SPSS, version 19). Data are presented as Mean ± Standard Deviation.

## 4. Results

The baseline characteristics of participants at day 0 are shown in Table 1. These characteristics, including age, weight, height, waist, BMI, FMD, TC, TG, LDL, HDL, FBS, apoA, apoB, VCAM, hsCRP, IL-6, and E-selectin, were compared between the CFOJ and FCOJ groups. There were no statistically significant differences between the characteristics of two groups at baseline (*P* ^$^ > 0.05).

The clinical characteristics of the 21 subjects that completed the study are displayed in Table 2. Consumption of both juices significantly decreased VCAM (*P* ^$^ < 0.001), hs-CRP (*P* ^$^ < 0.001), and E-selectin (*P* ^$^ < 0.001) but significantly increased apoA-1 (in COJ (*P* ^$^ = 0.019) and FOJ (*P* ^$^ = 0.003)). A significant decline in LDL occurred in the FOJ group (104.50 ± 27.26 to 99.18 ± 33.46 mg/dL, *P* ^$^ = 0.049).

There were no statistically significant differences between the characteristics of two groups (*P* ^@^ > 0.05), with the exception of apo A-1 levels that were significantly increased with both forms of OJ (for FOJ 117.05 ± 22.49 to 132.23 ± 13.09 mg/dL, *P* ^$^ = 0.003, and for COJ 116.95 ± 18.18 to 126.87 ± 23.01 mg/dL, *P* ^$^ = 0.019 (*P* ^@^ < 0.001)).

The percentage variation for each biochemical factor in each OJ group is shown in Table 3. The largest variations occurred with hs-CRP, VCAM in both juice groups.

## 5. Discussion

We used a single blind crossover randomized clinical trial to study the effects of COJ and FOJ on endothelial function by measuring FMD and inflammatory markers. During the study period, there were no underlying diseases or changes in lifestyle, including body weight, which may have influenced the observed results. Our study shows that OJ affects the physiologic characteristics of healthy people but that there were differences when consuming COJ and FOJ. Diet supplementation with both juices significantly decreased VCAM, hs-CRP, and E-selectin and significantly increased apo A-1. There was a significant decline in LDL in the FOJ group, while consuming FOJ significantly increased apo A-1 levels.

Our results did not reveal changes in FMD. The effects of OJ consumption on FMD have been studied to a limited extent in previous studies. Morand and colleagues reported that daily consumption of 500 mL blond orange juice for 4 weeks significantly improved endothelial function as assessed by cutaneous acetylcholine microvascular reactivity using Doppler laser flow. This effect was observed in the postprandial phase but not under fasting conditions [22]. It is unclear whether anthocyanins have reduced bioavailability due to poor absorption [27] or whether absorption is underestimated because of difficulties in the identification and measurement of all anthocyanin metabolites [28]. Another study tested the effects of cranberry juice (which contains a significant amount of anthocyanins) in subjects with coronary artery disease [24]. Although there was a reduction in the carotid femoral pulse wave velocity (a measure of arterial stiffness), brachial artery FMD did not change.

We also provide evidence that OJ had significant anti-inflammatory effects that resulted in a reduction of hs-CRP concentrations, which is a sensitive marker of inflammatory activation of the vessel wall and a frequently used biomarker of CVD [29]. The reduction of hs-CRP concentrations seemed to be of particular interest because it has been reported that hs-CRP concentrations of 2 mg/L are associated with a 30% reduction in risk of cardiovascular events [30]. The concentrations of inflammatory cytokines measured in this study (IL-6 and TNF-*α*) were also reduced after OJ consumption. Therefore, the observed reduction of hs-CRP, IL-6, E-selectin, and VCAM concentrations may have dampened inflammatory signaling with potentially beneficial effects on atherosclerotic progression [31]. These results are in agreement with in vitro studies that showed an anti-inflammatory action of oranges (mediated by a reduced expression of intracellular adhesion molecule 1, monocyte chemoattractant protein-1, and IL-8) in normal human keratinocytes stimulated with c-interferon and histamine [32].

The synthesis of CRP and other acute-phase proteins by hepatocytes is stimulated by proinflammatory cytokines such as IL-6, IL-1, and TNF-*α*, the expressions of which are regulated by the activation of the proinflammatory transcription factor nuclear transcription factor *κ*B (NF-*κΒ*) [33] that is mediated by oxidative stress. OJ contains antioxidants such as anthocyanins that act as ROS scavengers, an effect that in turn can potentially interfere with transcription factors such as NF-*κΒ*. Other potential mechanisms include the modulation of antioxidant gene transcription via the nuclear factor-erythroid 2 p45-related factor 2 (Nrf2) signaling pathway [34]. Studies investigating the role of anthocyanins in the activation of the Nrf2 pathway are sparse and essentially consist of in vitro experiments on the mechanisms that prevent hepatotoxicity. For example, Hwang et al. [35] reported that anthocyanins induced protective and anti-inflammatory effects on rat hepatocytes and inhibited the NF-*κΒ* and activated Nrf2 pathways; it cannot be excluded that both pathways are also involved in vivo, but this hypothesis remains to be investigated.

Levels of oxidative stress biomarkers such as 8-iso-prostaglandin are decreased after consumption of OJ [36] or other polyphenol-rich beverages [37]. Free radicals and reactive oxygen and nitrogen species react with proteins to lead to the conversion of amino acid residues into carbonyl groups. Therefore, protein carbonyl group (PC) concentrations are considered biomarkers of oxidative stress [38], suggesting that CVD patients should have higher serum PC concentrations. Consumption of OJ produced nonsignificant trends in reducing PC concentrations. It is possible that our study was underpowered for this variable, and a larger study may confirm this trend. It remains to be seen if higher OJ doses or longer periods of OJ intake might induce significant changes in PC concentrations.

## 6. Limitations

Important intrinsic limitations include the different taste characteristics of the two forms of OJ, making it difficult to design an improved double-blind study. It is unlikely though that prior knowledge of the kind of beverage ingested by the study participants could easily influence FMD or concentrations of inflammatory molecules. Longer treatment and washout periods were avoided to reduce subject burden and limit occurrence of other interacting factors such as infections, spontaneous fluctuations in diet, and physical activity. A carryover effect could not be formally excluded because of the short washout period and the lack of sampling at the start of the second period. The shapes of individual FMD trajectories argued against a significant carryover effect of OJ.

Both FOJ and COJ have relatively high levels of sugar, which can induce at least a mild form of insulin resistance and so alter Apo B and LDL levels. However, our studies were of an acute nature with little evidence of insulin resistance under these conditions as indicated by unchanged BMI and FBS values.

## 7. Conclusions

To the best of our knowledge, this is the first study to compare the effects of different types of OJ on inflammatory markers using a randomized, crossover, single-blind design. We observed favorable changes in some of inflammatory markers after the consumption of OJ in participants. Both COJ and FOJ positively affected physiological characteristics of healthy volunteers. Although these results may encourage the consumption of oranges, intervention studies are needed to test the effects of these types of OJ on metabolic and cardiovascular endpoints after prolonged consumption.

## Figures and Tables

**Table 1:** Main characteristics (Mean ± Standard Deviation) of participants at the baseline (day 0).

Characteristic	Group		*P*-value^$^
	COJ first	FOJ first	
Age (year)	34 ± 11	35 ± 12	0.769
Weight (kg)	62.70 ± 10.53	70.09 ± 9.80	0.112
Height (cm)	161.18 ± 10.07	169.27 ± 9.83	0.071
Waist (cm)	87.70 ± 9.51	89.64 ± 9.96	0.655
BMI (kg/m^2^)	24.40 ± 5.48	24.65 ± 4.29	0.905
FMD (%)	3.90 ± 1.10	3.27 ± 0.78	0.146
TC (mg/dL)	163.20 ± 36.75	179.09 ± 50.89	0.426
TG (mg/dL)	86.80 ± 35.28	159.09 ± 150.53	0.156
LDL (mg/dL)	100.10 ± 25.85	104.27 ± 33.71	0.756
HDL (mg/dL)	52.20 ± 7.87	44.55 ± 9.28	0.057
FBS (mg/dL)	84.27 ± 8.08	86.36 ± 7.72	0.542
Apo A-1 (mg/dL)	121.09 ± 20.69	122.91 ± 9.50	0.794
Apo B (mg/dL)	68.76 ± 16.94	73.73 ± 20.06	0.538
VCAM (ng/mL)	791.27 ± 281.37	647.91 ± 170.73	0.164
hsCRP (mg/dL)	1.05 ± 0.89	0.95 ± 0.67	0.770
IL6 (ng/mL)	6.83 ± 5.26	6.29 ± 4.77	0.808
E-selectin (ng/mL)	22.43 ± 9.77	27.84 ± 8.73	0.196

COJ: Commercial Orange Juice; FOJ: Fresh Orange Juice; Data expressed as ^$^ *P-*value for unpaired *t*-test; BMI: Body Mass Index; TC: Total cholesterol; TG: Triglyceride; LDL: Low-density lipoprotein; HDL: High-density lipoprotein; FBS: Fasting blood sugar.

**Table 2:** The effect of fresh and commercial orange juices on characteristics (PROC MIXED) of participants.

Characteristic	Before FOJ	After FOJ	*P*-value^$^	Before COJ	After COJ	*P*-value^$^	*P*-value^@^
Weight (kg)	67.64 ± 11.30	68.27 ± 11.22	0.066	66.38 ± 10.65	66.62 ± 10.32	0.309	0.999
Waist (cm)	88.36 ± 11.40	88.86 ± 11.12	0.352	88.95 ± 9.65	87.67 ± 9.91	0.053	0.282
BMI (kg/m^2^)	25.03 ± 5.18	25.27 ± 5.21	0.066	24.46 ± 4.27	24.55 ± 4.68	0.315	0.946
FMD (%)	3.31 ± 0.08	3.31 ± 0.16	1.00	3.57 ± 0.97	3.57 ± 0.97	1.00	0.887
TC (mg/dL)	181.32 ± 38.72	174.64 ± 42.18	0.091	170.19 ± 45.83	172.67 ± 41.04	0.698	0.066
TG (mg/dL)	126.14 ± 112.91	105.68 ± 73.11	0.153	112.81 ± 114.63	107.48 ± 91.04	0.514	0.201
LDL (mg/dL)	104.50 ± 27.26	99.18 ± 33.46	**0.049**	102.14 ± 33.12	97.71 ± 32.58	0.209	0.307
HDL (mg/dL)	49.64 ± 11.46	48.64 ± 16.22	0.751	48.76 ± 9.71	46.48 ± 11.61	0.394	0.901
FBS (mg/dL)	87.86 ± 7.05	91.95 ± 9.72	0.071	88.59 ± 18.28	88.38 ± 11.36	0.888	0.293
Apo A-1 (mg/dL)	117.05 ± 22.49	132.23 ± 13.09	**0.003**	116.95 ± 18.18	126.87 ± 23.01	0.019	**<0.001**
Apo B (mg/dL)	73.86 ± 21.02	69.50 ± 18.88	0.213	70.34 ± 17.11	67.67 ± 19.45	0.377	0.977
VCAM (ng/mL)	689.05 ± 254.52	533.82 ± 265.88	**<0.001**	693.82 ± 241.52	532.90 ± 222.47	**<0.001**	0.687
hsCRP (ng/ml)	1.06 ± 0.93	0.66 ± 0.63	**<0.001**	0.89 ± 0.72	0.56 ± 0.38	**0.011**	0.072
IL 6 (ng/mL)	6.68 ± 6.36	6.59 ± 4.99	0.954	6.40 ± 4.41	7.93 ± 8.03	0.311	0.452
E-selectin (ng/mL)	24.87 ± 9.20	17.79 ± 6.34	**<0.00**	24.70 ± 10.17	18.60 ± 7.45	**<0.001**	0.186

^$^ *P*-value computed for pair sample *t*-test. ^@^ *P*-value computed from PROC MIXED SAS software.

COJ: Commercial Orange Juice; FOJ: Fresh Orange Juice; FMD: Flow Mediated Dilation; SBP: Systolic Blood Pressure; DBP: Diastolic Blood Pressure; TC: Total Cholesterol; TG: Triglyceride; LDL: Low Density Lipoprotein; HDL: High Density Lipoprotein; FBS: Fasting Blood Sugar; Bold *P*-values are significant (*P* < 0.05).

**Table 3:** Variation percentage of characteristics after intervention compared to baseline in two study groups.

Characteristic	COJ Users (*N* = 22)	FOJ Users (*N* = 22)
FMD	0	0
TC (mg/dL)	−1.45	−3.69
TG (mg/dL)	−4.73	−16.22
LDL (mg/dL)	−4.34	−5.09
HDL (mg/dL)	4.91	2.05
FBS (mg/dL)	0.32	4.66
Apo A-1 (mg/dl)	9.37	12.97
Apo B (mg/dl)	−2.90	−5.91
VCAM (ng/mL)	−20.50	−22.53
hsCRP (ng/mL)	−35.00	−37.07
IL6 (ng/mL)	2.38	−1.41
E-selectin (ng/mL)	−24.61	−28.62

+: Increase; −: Decrease; COJ: Commercial Orange Juice; FOJ: Fresh Orange Juice.

## References

[1] Amiri M, Janssen F, Kunst AE (2011). The decline in ischaemic heart disease mortality in seven European countries: exploration of future trends. *Journal of Epidemiology and Community Health*.

[2] Amiri M, Kunst AE, Janssen F, Mackenbach JP (2006). Cohort-specific trends in stroke mortality in seven European countries were related to infant mortality rates. *Journal of Clinical Epidemiology*.

[3] Kunst AE, Amiri M, Janssen F (2011). The decline in stroke mortality: exploration of future trends in 7 Western European Countries. *Stroke*.

[4] Deanfield JE, Halcox JP, Rabelink TJ (2007). Endothelial function and dysfunction: testing and clinical relevance. *Circulation*.

[5] Furchgott RF, Zawadzki JV (1980). The obligatory role of endothelial cells in the relaxation of arterial smooth muscle by acetylcholine. *Nature*.

[6] Rossi R, Nuzzo A, Origliani G, Modena MG (2008). Prognostic role of flow-mediated dilation and cardiac risk factors in post-menopausal women. *Journal of the American College of Cardiology*.

[7] Yeboah J, Crouse JR, Hsu FC, Burke GL, Herrington DM (2007). Brachial flow-mediated dilation predicts incident cardiovascular events in older adults: the cardiovascular health study. *Circulation*.

[8] Shimabukuro M, Chinen I, Higa N, Takasu N, Yamakawa K, Ueda S (2007). Effects of dietary composition on postprandial endothelial function and adiponectin concentrations in healthy humans: a crossover controlled study. *The American Journal of Clinical Nutrition*.

[9] Ross R (1999). Atherosclerosis—an inflammatory disease. *The New England Journal of Medicine*.

[10] Buscemi S, Rosafio G, Arcoleo G (2012). Effects of red orange juice intake on endothelial function and inflammatory markers in adult subjects with increased cardiovascular risk. *The American Journal of Clinical Nutrition*.

[11] Chen L, Hu FB, Yeung E, Tobias DK, Willett WC, Zhang C (2012). Prepregnancy consumption of fruits and fruit juices and the risk of gestational diabetes mellitus: a prospective cohort study. *Diabetes Care*.

[12] Rohrmann S, Giovannucci E, Willett WC, Platz EA (2007). Fruit and vegetable consumption, intake of micronutrients, and benign prostatic hyperplasia in US men. *The American Journal of Clinical Nutrition*.

[13] Esmaillzadeh A, Kimiagar M, Mehrabi Y, Azadbakht L, Hu FB, Willett WC (2006). Fruit and vegetable intakes, C-reactive protein, and the metabolic syndrome. *The American Journal of Clinical Nutrition*.

[14] Tsai CJ, Leitzmann MF, Willett WC, Giovannucci EL (2006). Fruit and vegetable consumption and risk of cholecystectomy in women. *The American Journal of Medicine*.

[15] Michels KB, Giovannucci E, Chan AT, Singhania R, Fuchs CS, Willett WC (2006). Fruit and vegetable consumption and colorectal adenomas in the nurses’ health study. *Cancer Research*.

[16] Hooper L, Kroon PA, Rimm EB (2008). Flavonoids, flavonoid-rich foods, and cardiovascular risk: a meta-analysis of randomized controlled trials. *The American Journal of Clinical Nutrition*.

[17] Johnsen SP, Overvad K, Stripp C, Tjønneland A, Husted SE, Sørensen HT (2003). Intake of fruit and vegetables and the risk of ischemic stroke in a cohort of Danish men and women. *The American Journal of Clinical Nutrition*.

[18] Dauchet L, Amouyel P, Hercberg S, Dallongeville J (2006). Fruit and vegetable consumption and risk of coronary heart disease: a meta-analysis of cohort studies. *Journal of Nutrition*.

[19] Cesar TB, Aptekmann NP, Araujo MP, Vinagre CC, Maranhão RC (2010). Orange juice decreases low-density lipoprotein cholesterol in hypercholesterolemic subjects and improves lipid transfer to high-density lipoprotein in normal and hypercholesterolemic subjects. *Nutrition Research*.

[20] Ghanim H, Mohanty P, Pathak R, Chaudhuri A, Chang LS, Dandona P (2007). Orange juice or fructose intake does not induce oxidative and inflammatory response. *Diabetes Care*.

[21] USDA: United States Department of Agriculture Database for the flavonoid content of selected foods. http://www.ars.usda.gov/Services/docs.htm?docid=6231.

[22] Morand C, Dubray C, Milenkovic D (2011). Hesperidin contributes to the vascular protective effects of orange juice: a randomized crossover study in healthy volunteers. *The American Journal of Clinical Nutrition*.

[23] Riso P, Visioli F, Gardana C (2005). Effects of blood orange juice intake on antioxidant bioavailability and on different markers related to oxidative stress. *Journal of Agricultural and Food Chemistry*.

[24] Dohadwala MM, Holbrook M, Hamburg NM (2011). Effects of cranberry juice consumption on vascular function in patients with coronary artery disease. *The American Journal of Clinical Nutrition*.

[25] World Health Organization (1998). *Prevention and Management of the Global Epidemic of Obesity*.

[26] Corretti MC, Anderson TJ, Benjamin EJ (2002). Guidelines for the ultrasound assessment of endothelial-dependent flow-mediated vasodilation of the brachial artery: a report of the international brachial artery reactivity task force. *Journal of the American College of Cardiology*.

[27] Prior RL, Meskin MS, Bidlack WR, Davies AJ, Lewis DS, Randolph RK (2004). Absorption and metabolism of anthocyanins: potential health effects. *Phytochemicals Mechanisms of Action*.

[28] Milbury PE, Vita JA, Blumberg JB (2010). Anthocyanins are bioavailable in humans following an acute dose of cranberry juice. *Journal of Nutrition*.

[29] Libby P (2006). Inflammation and cardiovascular disease mechanisms. *The American Journal of Clinical Nutrition*.

[30] Ridker PM, Cannon CP, Morrow D (2005). C-reactive protein levels and outcomes after statin therapy. *The New England Journal of Medicine*.

[31] Wilson AM, Swan JD, Ding H (2007). Widespread vascular production of C-reactive protein (CRP) and a relationship between serum CRP, plaque CRP and intimal hypertrophy. *Atherosclerosis*.

[32] Cardile V, Frasca G, Rizza L, Rapisarda P, Bonina F (2010). Antiinflammatory effects of a red orange extract in human keratinocytes treated with interferon-gamma and histamine. *Phytotherapy Research*.

[33] de Winther MP, Kanters E, Kraal G, Hofker MH (2005). Nuclear factor *κ*B signaling in atherogenesis. *Arteriosclerosis, Thrombosis, and Vascular Biology*.

[34] Mann GE, Rowlands DJ, Li FYL, de Winter P, Siow RCM (2007). Activation of endothelial nitric oxide synthase by dietary isoflavones: role of NO in Nrf2-mediated antioxidant gene expression. *Cardiovascular Research*.

[35] Hwang YP, Choi JH, Yun HJ (2011). Anthocyanins from purple sweet potato attenuate dimethylnitrosamine-induced liver injury in rats by inducing Nrf2-mediated antioxidant enzymes and reducing COX-2 and iNOS expression. *Food and Chemical Toxicology*.

[36] Sánchez-Moreno C, Cano MP, de Ancos B (2003). Effect of orange juice intake on vitamin C concentrations and biomarkers of antioxidant status in humans. *The American Journal of Clinical Nutrition*.

[37] Nemzer BV, Rodriguez LC, Hammond L, Disilvestro R, Hunter JM, Pietrzkowski Z (2011). Acute reduction of serum 8-iso-PGF2-alpha and advanced oxidation protein products in vivo by a polyphenol-rich beverage; a pilot clinical study with phytochemical and in vitro antioxidant characterization. *Nutrition Journal*.

[38] Dalle-Donne I, Rossi R, Giustarini D, Milzani A, Colombo R (2003). Protein carbonyl groups as biomarkers of oxidative stress. *Clinica Chimica Acta*.

